# Paradigm Shift in the Management of the Atrophic Posterior Maxilla

**DOI:** 10.1155/2014/486949

**Published:** 2014-11-10

**Authors:** Rabah Nedir, Nathalie Nurdin, Paul Khoury, Marc El Hage, Semaan Abi Najm, Mark Bischof

**Affiliations:** ^1^Ardentis Clinique Dentaire Vevey, Swiss Dental Clinics Group, Rue du Collège 3, 1800 Vevey, Switzerland; ^2^Department of Oral and Maxillofacial Surgery, Oral Surgery and Implantology Unit, Geneva University Hospitals, Rue Barthélemy-Menn 19, 1205 Geneva, Switzerland; ^3^Ardentis Clinique Dentaire Lausanne, Swiss Dental Clinics Group, Voie du Chariot 6, 1003 Lausanne, Switzerland; ^4^Ardentis Clinique Dentaire Geneva, Swiss Dental Clinics Group, Rue Thomas-Masaryk 1, 1202 Geneva, Switzerland

## Abstract

When the posterior maxilla is atrophic, the reference standard of care would be to perform sinus augmentation with an autologous bone graft through the lateral approach and delayed implant placement. However, placement of short implants with the osteotome sinus floor elevation technique and without graft can be proposed for an efficient treatment of clinical cases with a maxillary residual bone height of 4 to 8 mm. The use of grafting material is recommended only when the residual bone height is ≤4 mm. Indications of the lateral sinus floor elevation are limited to cases with a residual bone height ≤ 2 mm and fused corticals, uncompleted healing of the edentulous site, and absence of flat cortical bone crest or when the patient wishes to wear a removable prosthesis during the healing period. The presented case report illustrates osteotome sinus floor elevation with and without grafting and simultaneous implant placement in extreme conditions: atrophic maxilla, short implant placement, reduced healing time, and single crown rehabilitation. After 6 years, all placed implants were functional with an endosinus bone gain.

## 1. Introduction

The posterior maxilla remains a challenging area for implant-supported rehabilitation. After teeth loss, alveolar bone resorbs and pneumatization of the maxillary sinus happens at the expense of the residual bone height (RBH); the majority of edentulous molar sites exhibit an RBH < 7 mm [1].

The two main techniques to augment the sinus floor height are the lateral sinus floor elevation (LSFE or sinus-lift technique) and the osteotome sinus floor elevation via a crestal approach (OSFE). First introduced by Boyne and James [2], the LSFE procedure schematically involves access of the sinus cavity via a bony window created in its lateral wall. The Schneiderian membrane is elevated creating a confined space into which a grafting material is placed. The window is then covered with a barrier membrane and the flap is sutured over [3]. A healing period of 4 to 12 months is respected before placing the implants. This procedure has a predictable long-term success rate which can reach 96.5% using implants with rough surface [4]. However, it depends highly on the skill and experience of the surgeon, even though the sinus membrane is elevated under direct visual control. The procedure is time-consuming and invasive, with additional morbidity related to the harvesting of autogenous bone from a donor site. The incidence of graft failure ranges from 0 to 17.9% [5].

In 1994, Summers suggested a crestal approach to augment the maxillary bone height locally with sinus osteotomes [6]. A single access site is prepared through the crestal bone. The sinus floor is locally fractured and the Schneiderian membrane is apically internally elevated, using a series of osteotomes of increasing diameter. The grafting material is then inserted into the prepared site and the implant is simultaneously placed, in a single surgical procedure. The use of osteotomes condenses progressively the adjacent residual bone and consequently increases its density. Additionally, higher bone-implant contact is obtained [7]. However, when primary stability is poor, implant placement must be delayed. The OSFE technique yields predictable results, with success rates reaching 95% [8–10]. It limits the extent of the surgical site through a minimal invasive surgery and therefore postoperative discomfort is attenuated and is comparable to that of standard implant placement. Although this procedure is less invasive and faster than the LSFE technique, it is also technically demanding.

Actually, both patients and practitioners demand simplified predictable approach to increase acceptance of implant placement. For some authors, LSFE is not indicated in cases of RBH between 4 and 6 mm. As an RBH of 4 mm can ensure implant primary stability and osseointegration, the indication of the OSFE has been extended to this RBH range [11–13]. However, the achievement of primary stability is still challenging when the RBH is ≤4 mm. Standard cylindrical implants, with a large thread pitch, do not reach primary stability in limited bone height because they cannot engage bone more than a single row of threads. Tapered implants have been designed with a cylindroconical shape and a reduced thread pitch. Through compressive insertion, they achieve sufficient primary stability in soft bone [14] and in sites of maxillary RBH as low as 2 mm [11]. Localized membrane elevation and implant placement are carried out simultaneously in one stage.

Grafting material may not be required when limited augmentation of the sinus floor is needed [11, 15–17]. Elevation of the Schneiderian membrane creates a compartment in which the blood clot is lodged. The stabilized blood clot has the potential to stimulate bone formation [16]. The absence of grafting material eliminates the risks that result from a secondary surgical procedure at the donor site (infection, surgical trauma) and overfilling of the maxillary sinus (necrosis of the membrane, loss of the graft into the sinus, and sinusitis) [18]. It reduces surgical cost and the healing period is shortened.

## 2. Case Report

The following case illustrates OSFE and simultaneous short tapered implant placement in very atrophic maxilla, with and without grafting material.

A 58-year-old nonsmoker Caucasian man presented for rehabilitation of his left and right atrophic posterior maxilla that had been edentulous for five years. He required implant placement to support a fixed prosthesis with the least invasive and shortest procedure. His general health was good without a contributive medical history. The RBH was measured on an orthopantomograph before surgery. The preoperative subsinus mean RBH was 1.6 mm at the right sinus (sites 16 and 17) and 2.2 mm at the left sinus (sites 26 and 27). Preoperative cone-beam computed tomography (CBCT) was not necessary.

**Figure 1:**
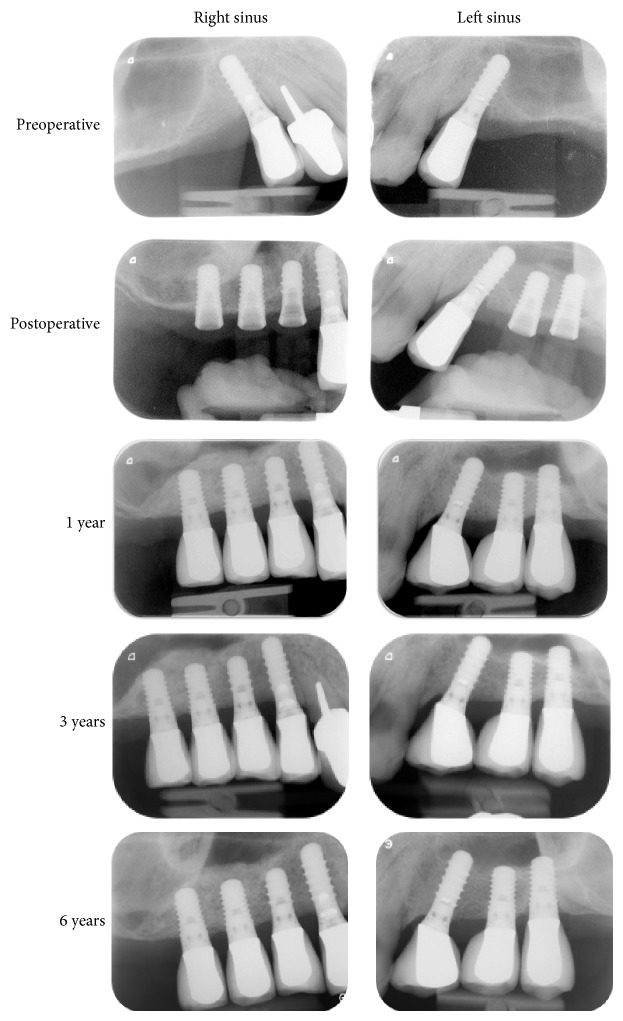
Periapical radiographs. The 8 mm tapered implants were placed using the osteotome sinus floor elevation procedure with grafting material (right sinus) and without grafting material (left sinus). In all controls after surgery, implants were clinically stable. A radioopaque area corresponding to the augmented sinus floor was present around the implants placed without graft and around and above the implants placed with graft.

**Figure 2:**
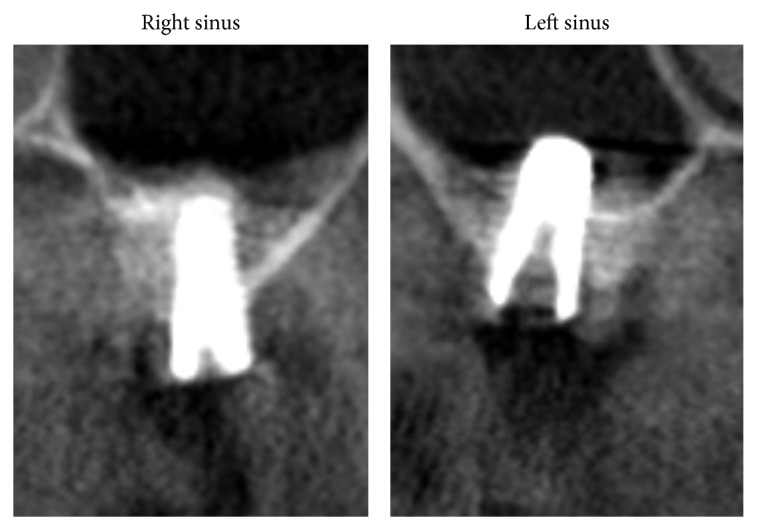
CBCT exam at 5 years. Note the absence of Schneiderian membrane thickening on the coronal view of implant placed with grafting material (right sinus, second molar site) and without grafting material (left sinus, second molar site). The CBCT confirms that the implant in the left sinus protruded into the sinus whereas the implant in the right sinus was completely embedded in peri-implant bone.

The surgical procedures were carried out under antibiotic prophylaxis initiated the day prior to surgery (Amoxi-Mepha, Mepha Pharma SA, Aesch, Basel, Switzerland; 750 mg, 3 x/day for 6 days). On both sides, a midcrestal incision was performed for flap elevation, without any vertical or periosteal releasing incision. The cortical bone was marked using round burs of increasing diameter (*Ø* 1.4–3.1 mm). Whatever the bone density, a *Ø* 2.8 mm sinus floor elevation osteotome (Straumann AG, Basel, Switzerland) was first utilized. Careful light tapping with a mallet pushed the bony sinus floor into the sinus cavity; this elevated the Schneiderian membrane. The osteotomy site was then enlarged with the Ø 3.5 mm osteotome; integrity of the membrane was controlled with an undersized Ø 2.1 mm depth gauge and by implementing the Valsalva maneuver. The elevated right sinus was filled with 0.5 cm^3^ (0.25 g) of Bio-Oss (Geistlich Pharma AG, Wolhusen, Switzerland; granulometry 0.25–1 mm) and two TE SLActive implants (Straumann AG, Basel, Switzerland; *Ø* 4.1/4.8 mm, length 8 mm) were placed without bone tapping (sites 16 and 17). In the left sinus, two TE SLActive implants (*Ø* 4.1/4.8 mm, length 8 mm) were placed without grafting material and without bone tapping (sites 26 and 27). All implants were seated in the osteotomy site until the rough surface limit was no longer visible on the mesial and distal sides; implant neck was protruding above the crest. Implants were left to heal transgingivally; the sites were kept prosthesis-free over the whole healing period. The healing period was uneventful. Three months after surgery, implants were osteointegrated and screw-retained porcelain-fused-to-gold single crowns were screwed into the implants and functionally loaded. Standardized periapical radiographs were taken immediately after surgery, at 1, 3, and 5 years with implant suprastructure unscrewed. Endosinus bone height between the most coronal implant thread and the most apical implant-bone contact was measured along both sides of each implant; it was then averaged. An increase of this mean value on consecutive radiographs was indicative of endosinus bone gain. Periapical radiographs with crowns were taken at 1, 3, and 6 years (Figure 1). The 5-year control included a CBCT analysis (Figure 2).

After 6 years, all implants yielded successful results, even those placed without graft. No failed implants were recorded. The 5-year CBCT analysis shows no thickening of the mucosa for both sinuses. After 1 year, the mean endosinus bone gain was 5.1 mm around implants placed without grafting material and 6.8 mm around implants placed with grafting material. After 5 years, it was 5.3 mm and 6.8 mm, respectively. The implants without grafting were protruded within the sinus and covered by the sinus membrane. All implants placed with grafting material were entirely embedded in the newly formed mineralized tissue. The mean height of the bony dome was 1.4 mm at 1 year; the bony dome remained stable over 5 years.

## 3. Discussion

The classical therapeutic options for the treatment of the edentulous posterior maxilla are dictated by the available RBH and implant length. They comprise

- Procedure A: standard implant placement;
- Procedure B: sinus augmentation using a crestal approach with sinus osteotomes and simultaneous implant placement;
- Procedure C: sinus augmentation using a lateral approach and simultaneous implant placement;
- Procedure D: sinus augmentation using a lateral approach and delayed implant placement.

A consensus conference held in 1996 on sinus lifting recommended the selection of the surgical approach according to the RBH [5]. When using implants ≥ 10 mm in length,

- Procedure A is applied when RBH is ≥10 mm;
- Procedure B is applied when RBH ranges from 7 to 9 mm;
- Procedure C is applied when RBH ranges from 4 to 6 mm;
- Procedure D is applied when RBH ranges from 1 to 3 mm.

Autogenous bone graft has been the most widely used grafting material [19]. To avoid the problems related to bone harvesting at secondary surgical sites, bone substitutes were proposed. They can fill large volumes. Xenograft materials of bovine origin like Bio-Oss showed no osteoinductive potential, but osteoconductive properties; they act as a scaffold for new bone apposition [20]. They are most often used and are well documented [21]. The use of alloplastic materials is also possible. This is offered to patients who are averse or reluctant to the use of animal-derived products. Some fully synthetic materials, such as synthetic hydroxyapatite and tricalcium phosphate, have shown good osteoconduction properties with specific resorption rates [22]. They are promising but need further and larger studies in comparison with Bio-Oss. The choice of the most suitable grafting material for sinus augmentation has been a subject of controversy for a long time; however, in the recent years, the need to use grafting material for sinus augmentation has been questioned [23].

The implant/abutment interface determines joint strength, stability, and lateral and rotational stability. The implant/abutment connection is generally described as internal or external. Historically, Branemark's original implant-abutment connection was an external hexagon. It was developed for the restoration of completely edentulous arches using a series of implants connected to one another with a metal bar. However, significant complications occurred when it was used in other indications, such as fixed partial denture and single tooth replacement in the posterior maxilla. To overcome these complications, internal connection implants were designed. The vertical height platform for restorative components was hence reduced. This improves connection stability throughout function and placement and simplifies restoration [24]. Therefore, by inducing a high mechanical strength, the use of internally connected implants is more appropriate for rehabilitation of the posterior maxilla.

The standard current clinical practice to treat the presented case would have required a sinus-lift procedure with graft insertion and delayed implant placement [5]. The procedure selected involved OSFE and simultaneous placement of 8 mm long tapered implants with a reduced thread pitch. This procedure avoids invasive surgery and permits treatment within a single surgical step with a reduced healing time. Implants were placed with good primary stability in the atrophic maxillary bone. In the extreme conditions reported here, very low RBH, use of tapered short implants, reduced healing time, and single crown rehabilitation, both the* de novo* bone and the composite regenerated mineralized tissue showed an ability to support functional implants. After 6 years, the implants demonstrated successful integration and occlusal loading.

The OSFE technique performed with graft led to a complete embedding of the implants with a mean endosinus bone gain of 6.8 mm after 5 years. The OSFE technique without grafting material allowed increasing the bone height around the implants of 5.3 mm on average. Previous publications reported that consistent endosinus bone gain can be achieved without any grafting material. In a study by Nedir et al., 17 patients received 25 implants in a mean RBH of 5.4 ± 2.3 mm [11]; after 1 year, all sites showed endosinus bone gain (mean gain 2.5 ± 1.2 mm). The regenerated volume did not shrink with time but rather increased to 3.2 ± 1.3 mm after 5 years, with a predictable implant success for up to 5 years [25, 26]. Lai et al. observed a 5-year cumulative survival rate of 97.4% for implants placed in a mean RBH of 5.6 ± 2.5 mm [27]. Endosinus bone gain was 2.66 ± 0.87 mm at the 9-month follow-up examination. The first published randomized study comparing the behaviors of tapered implants placed with and without grafting material in atrophic posterior maxillae (RBH ≤ 4 mm; range: 0.9–4.0 mm) showed that both OSFE procedures were efficient [28]. However, complications occurred when implants were placed in sites with a fused cortical bone (“monocortical bone”). When a grafting material was used, more bone was obtained (mean bone gain at the 1-year time point with grafting: 5.0 ± 1.3 mm; without grafting: 3.9 ± 1.0 mm). Another study reported favorable results in a 3-year follow-up of implants randomly placed with and without grafting material in bone with a mean RBH of 4.6 ± 1.3 mm (range: 2–8 mm) [29]. The cumulative survival rate was 95.1%. The mean 3-year bone gain reached 3 mm for both implant groups.

It was reported that implant failures occur more frequently when using short implants than long ones [30]. The reasons put forward include insufficient bone-to-implant contact and unfavorable crown-to-implant ratio [31]. However, some studies showed that rough implants less than 10 mm in length yielded successful outcomes even when placed in atrophic maxillae with OSFE [31–36]. The use of short implants minimizes the extent of sinus floor elevation. It reduces thus the risk of membrane perforation and the need for large volumes of grafting material [30, 35]. Depending on the thickness and adherence of the membrane, sinus morphology, and the number of implants placed, the Schneiderian membrane can support an elevation of 4–8 mm [37]. Therefore, 8 mm implants can be surrounded by* de novo* bone sufficiently for implant anchorage, even without grafting material [28]. A bone anchorage height of 6.4 mm proved sufficient to ensure implant function in the posterior area after 3-year observation periods [38]. The utility of complete implant bone coverage has been questioned, as well as the presence of bone tissue or nonresorbed material above the implant apex.

**Figure 3:**
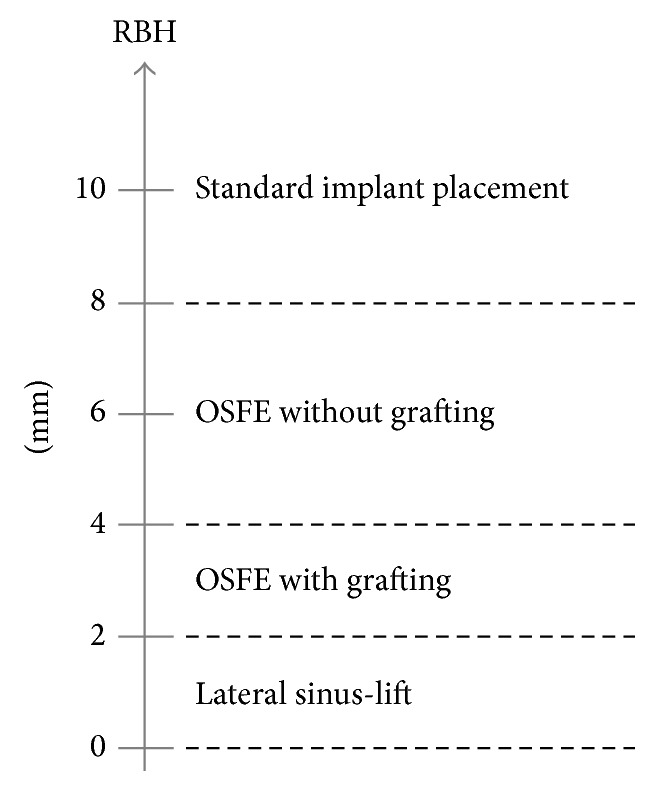
New concept for the management of the posterior maxilla using 8 mm tapered implants. The use of OSFE in patients with a maxillary RBH > 2 mm is facilitated by placing tapered implants with a reduced thread pitch. The lateral sinus-lift procedure, with delayed implant placement, is indicated when primary stability cannot be reached with OSFE and when a large amount of bone gain is required.

In light of the progress in the atrophic posterior maxilla management, a concept for an efficient treatment of this region is proposed (Figure 3).

- When the preoperative available RBH is >8 mm, standard 10 mm long implant placement is performed; implant penetration of 1-2 mm into the sinus is tolerated.
- The use of OSFE without grafting material is advocated for a maxillary RBH between 4 and 8 mm. It allows implants to be placed in one session without the need of preoperative CBCT. Absence of graft does not compromise the success of OSFE and implant survival; the regenerative properties of the bone within the sinus lead to marked endosinus bone formation. In the presence of residual bone with a radiographically distinct crestal cortical and sinus floor cortical bone, sufficient primary stability can be obtained using tapered implants with a reduced thread pitch. Insertion of short 8 or 10 mm implants is recommended, with one prosthetic element per implant. They further reduce the need for grafting because of limited protrusion.
- The sites with an RBH in the range of 2–4 mm can be successfully rehabilitated in using OSFE procedure associated with the simultaneous placement of 8 mm long implants. A bone regeneration height of at least 3-4 mm along the 8 mm implant renders the LSFE unnecessary; the LSFE procedure is no more clinically indicated. The insertion of grafting material, in maintaining the Schneiderian membrane elevated, increases the endosinus bone formation around the implants. This improves peri-implant bone anchorage in such extreme situations.

The LSFE technique is exceptionally used; it is preferable when large bone gain is needed (RBH ≤ 2 mm) and when

- corticals are fused (monocortical bone) or absent: sinus and crestal cortical bone must be radiographically distinct and separated in order to insure an adequate primary stability when using OSFE;
- edentulous sites are not completely flat and healed before surgery: teeth extractions at the intended implant site must have been performed for at least four months before OSFE;
- the patient wishes to have a removable prosthesis at the implant sites during the healing period.

## 4. Conclusion

Increased acceptance of implant placement among patients requires a fast, minimally invasive, and predictable treatment. OSFE, with simultaneous placement of short tapered rough-surfaced implants with a reduced thread pitch, is a reliable treatment for most clinical indications in the posterior maxilla. This procedure shortens surgery and healing time and reduces treatment costs. It renders implant treatment accessible to a larger pool of patients. It may be used without grafting material in patients with a maxillary RBH > 4 mm or with grafting material in patients with an RBH of 2–4 mm. When the RBH is ≤2 mm, sinus elevation via a lateral approach with the use of grafting material is still recommended. Therefore, the LSFE technique can no longer be considered the standard procedure for implant-supported rehabilitation of the atrophic maxilla.
